# Defining Overweight and Obesity by Percent Body Fat Instead of Body Mass Index

**DOI:** 10.1210/clinem/dgae341

**Published:** 2024-05-15

**Authors:** Adam W Potter, Geoffrey C Chin, David P Looney, Karl E Friedl

**Affiliations:** Thermal and Mountain Medicine Division, U.S. Army Research Institute of Environmental Medicine, Natick, MA 01760-5007, USA; Thermal and Mountain Medicine Division, U.S. Army Research Institute of Environmental Medicine, Natick, MA 01760-5007, USA; Military Performance Division, U.S. Army Research Institute of Environmental Medicine, Natick, MA 01760-5007, USA; Office of the Senior Scientist, U.S. Army Research Institute of Environmental Medicine, Natick, MA 01760-5007, USA

**Keywords:** body fat, body mass index, BMI, metabolic syndrome

## Abstract

**Objective:**

Thresholds for overweight and obesity are currently defined by body mass index (BMI), a poor surrogate marker of actual adiposity (percent body fat [%BF]). Practical modern technologies provide estimates of %BF but medical providers need outcome-based %BF thresholds to guide patients. This analysis determines %BF thresholds based on key obesity-related comorbidities, exhibited as metabolic syndrome (MetSyn). These limits were compared to existing BMI thresholds of overweight and obesity.

**Design:**

Correlational analysis of data from cross sectional sampling of 16 918 adults (8734 men and 8184 women) from the US population, accessed by the National Health and Nutrition Examination Survey public use datasets.

**Results:**

Individuals measured by BMI as overweight (BMI > 25 kg/m^2^) and with obesity (BMI > 30 kg/m^2^) included 5% and 35% of individuals with MetSyn, respectively. For men, there were no cases of MetSyn below 18%BF, %BF equivalence to “overweight” (ie, 5% of MetSyn individuals) occurred at 25%BF, and “obesity” (ie, 35% of MetSyn individuals) corresponded to 30%BF. For women, there were no cases of MetSyn below 30%BF, “overweight” occurred at 36%BF, and “obesity” corresponded to 42%BF. Comparison of BMI to %BF illustrates the wide range of variability in BMI prediction of %BF, highlighting the potential importance of using more direct measures of adiposity to manage obesity-related disease.

**Conclusion:**

Practical methods of body composition estimation can now replace the indirect BMI assessment for obesity management, using threshold values provided from this study. Clinically relevant “overweight” can be defined as 25% and 36% BF for men and women, respectively, and “obesity” is defined as 30% and 42% BF for men and women.

Clinical health standards currently define thresholds for healthy, overweight, and obesity by body mass index (BMI; kg/m^2^). Although BMI has long been the primary metric in clinical weight management, it has also been well recognized to be a poor surrogate marker of actual adiposity or relative body fat (%BF) (1). Practical modern technologies are beginning to provide more reliable estimates of %BF but for these to be useful, medical providers require outcome-based %BF thresholds to help guide patient health. These %BF metrics to advise patients about obesity-related health risks require direct derivation from clinical health outcomes. Previous attempts to derive %BF health risk thresholds from BMI are problematic because of the imprecise relationship and the relationship is further affected by factors such as age, sex, nutrition, and fitness habits (2, 3).

Obesity-related diseases have been defined on the basis of excess adiposity, primarily via deposition of lipids in liver and muscle and decreases in whole-body insulin sensitivity (4). However, instead of targeting the association of %BF with obesity-related diseases, “ideal weight” recommendations for Americans have been generalized to associations with all-cause mortality. This strategy began with the 1912 height-weight tables that were derived from mortality statistics of the insured population, and it continued with later updates such as the Metropolitan Life ideal weight tables in 1959 and 1983 (5). In an attempt to check the rising prevalence of obesity, the 1985 National Institutes of Health Obesity Consensus panel simply provided arbitrary weight targets for the country, calculated from the 85th percentile of the BMI of men and women in the National Health and Nutrition Examination Survey (NHANES) II (27.8 and 27.3 kg/m^2^, respectively) (6). Finally, the 1998 National Heart, Lung, and Blood Institute expert panel developed the current definitions of “overweight” established as BMI > 25 kg/m^2^ and “obese” as BMI > 30 kg/m^2^, roughly based on BMI inflection points for various obesity-related disease risks. However, there is a continued focus on the association between BMI thresholds and all-cause mortality (7, 8). These thresholds were based on simple anthropometrics (BMI and waist circumference) because these were the only practical metrics available on which to base national health goals. The National Institutes of Health expert panel review of bioelectrical impedance technologies held out hope for the future maturity of practical methods for %BF estimation to more effectively target obesity disease management (9). Today, practical methods to estimate %BF, such as multifrequency bioelectrical impedance, are maturing and may find an important role in preventive medicine.

Metabolic syndrome (MetSyn), affecting an estimated one third of adult Americans, has a plausible mechanistic relationship to %BF (4, 10, 11). On this basis, the use of %BF metrics could be more useful than body size (BMI) in guiding patients because excess fat is either a key marker or a direct cause of metabolic disease. The Adult Treatment Panel III from the National Cholesterol Education Program, classified MetSyn when 3 or more of the 5 key markers are present. The Adult Treatment Panel III criteria used for MetSyn includes waist circumference >101.6 cm (men), >88.9 cm (women); high-density lipoprotein cholesterol < 40 mg/dL (men), <50 mg/dL (women); fasting glucose ≥ 100 mg/dL; blood pressure > 130/85 mm Hg; and triglycerides ≥ 150 mg/dL (12).

This analysis uses a large diverse sample of data to provide sex-specific thresholds of %BF based on key obesity-related comorbidities, exhibited as MetSyn. These upper limits of %BF to categorize men and women were compared to existing BMI thresholds of overweight and obesity. The concept was to derive %BF thresholds directly from the MetSyn outcomes instead of less directly by translation from BMI. The thresholds were established based on equivalent MetSyn population outcomes for the 2 commonly used BMI-based definitions of “overweight” and “obesity.” Use of these measures of %BF may be more clinically relevant markers for metabolic health across the general population than body size (ie, BMI).

## Materials and Methods

### Study Design and Sample Population

This is a correlational analysis of data from cross sectional sampling of the US population via the NHANES public use datasets (13). NHANES provides a demographically representative sampling of the US population that is collected continuously and collated into 2-year datasets.

Individual participant data were obtained from the NHANES (13, 14). The NHANES has been approved by the National Center for Health Statistics Research Ethics Review Board; therefore, the present analyses did not require a separate regulatory approval. Each participant within the NHANES study provided written informed consent before assessments (13).

**Figure 1.**
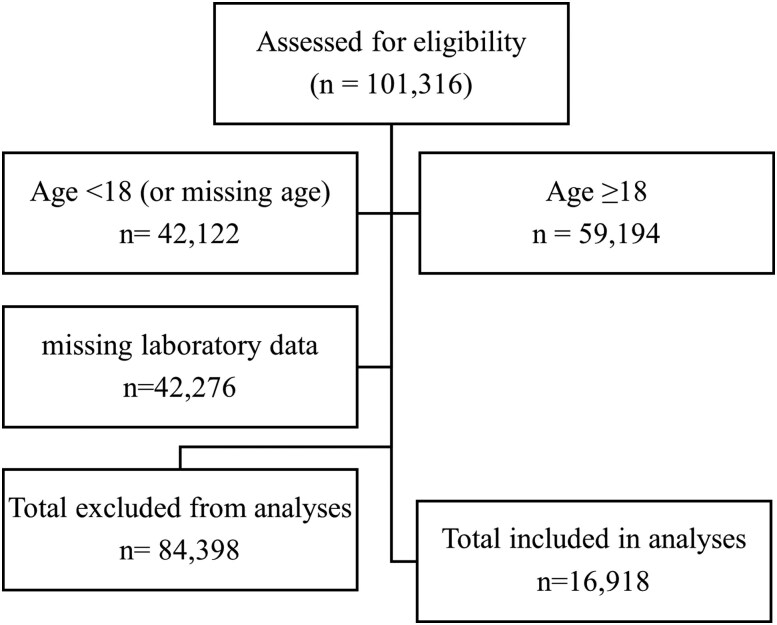
CONSORT flow diagram for study participant data selection.

The present analyses include data from 16 918 adults (8734 men and 8184 women), ages 18 to 85 years, from NHANES 1999-2018. Data were obtained for individual demographics (age, race/ethnicity), laboratory measures: fasting glucose (mg/dL), triglycerides (mg/dL), high-density lipoprotein-C (mg/dL), and blood pressure (mm Hg), body measures (height, weight, waist circumference, and BMI), and from whole-body dual-energy x-ray absorptiometry (DXA) assessments (Hologic, Inc., Bedford, Massachusetts). Individual data were included if associated DXA measures were available; as a result, two survey periods with no recorded DXA measures (2007-2008 and 2009-2010) were not included. For the present analysis, classification for metabolic health outcomes for each individual was made based on meeting the criteria for MetSyn (≥3 markers) (12). The CONSORT flow diagram is presented in Fig. 1.

### Statistical Analyses

Statistical analyses were conducted using a combination of SAS version 9.4 (SAS Institute), SPSS version 28.01.1 (IBM Corporation), and Excel (Microsoft Corporation). Descriptive statistics are shown as mean ± standard deviation or by number of incidences.

## Results

Data analyses were conducted on 16 918 adults (8734 men, mean age 41.6 years; 8184 women, mean age 42.6 years), including self-reported race/ethnicity as 39% non-Hispanic White, 27% Hispanic, 21% non-Hispanic Black, 8% non-Hispanic Asian, and 4% non-Hispanic multiple (0.4% no answer). Descriptive statistics are shown in Tables 1-3.

**Table 1.** Study descriptive statistics

	Men	Women
n	8734	8184
Age (y)	41.6 ± 16.5	42.6 ± 16.4
BMI (kg/m^2^)	28.7 ± 6.5	29.3 ± 7.7
LBM (kg)	62.7 ± 11.2	45.4 ± 9.2
BMC (kg)	2.64 ± 0.45	2.09 ± 0.36
BMD (cm^3^)	1.17 ± 0.12	1.08 ± 0.12
BF (%)	27.8 ± 6.5	39.7 ± 6.5
MetSyn (n,%)	3353 (38.4%)	2375 (29.0%)

Abbreviations: BF, body fat; BMC, bone mineral content; BMD, bone mineral density; BMI, body mass index; LBM, lean body mass; MetSyn, metabolic syndrome.

**Table 2.** Study sex and age group distribution

Age group (y)	Men	Women	Total (proportion %)	MetSyn cases (% of age group)
n	8734	8184	16 918	5728 (33.9%)
18-29	2435	2099	4534 (26.8%)	610 (13.5%)
30-39	1731	1532	3263 (19.3%)	920 (28.2%)
40-49	1765	1776	3541 (20.9%)	1319 (37.2%)
50-59	1615	1609	3224 (19.1%)	1454 (45.1%)
60-69	641	642	1283 (7.6%)	785 (61.2%)
70-79	343	293	636 (3.8%	408 (64.2%)
≥80	204	233	437 (2.6%)	232 (53.1%)

Abbreviation: MetSyn, metabolic syndrome.

**Table 3.** Study sex and race/ethnicity group distribution

	Men	Women	Total (proportion %)	MetSyn Cases (% of subgroup)
n	8734	8184	16 918	
Hispanic	2376	2254	4630 (27.4%)	1272 (38.8%)
Non-Hispanic White	3527	3164	6691 (39.5%)	456 (37.0%)
Non-Hispanic Black	1731	1790	3521 (20.8%)	2477 (28.7%)
Non-Hispanic Asian	697	628	1325 (7.8%)	246 (18.6%)
Non-Hispanic multiple	370	320	690 (4.1%)	217 (31.4%)
No answer	33	28	61 (0.4%)	51 (83.6%)

Abbreviation: MetSyn, metabolic syndrome.

### Redefined Overweight and Obesity on the Basis of Relative Body fat (%BF)

**Figure 2.**
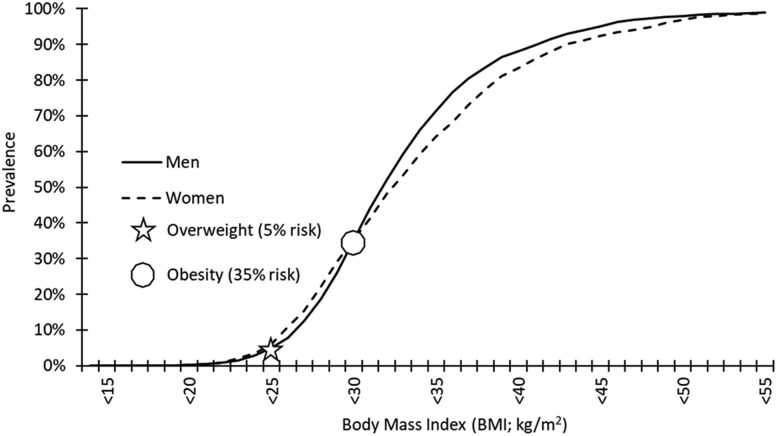
Prevalence of men and women in the NHANES data with ≥3 markers of metabolic syndrome by BMI.

The percentage of individuals with MetSyn at BMI-defined limits of overweight (BMI >25 kg/m^2^) and obesity (BMI ≥30 kg/m^2^) were 5% and 35% (Fig. 2). These rates of MetSyn were then used to define %BF thresholds for comparable definitions of %BF-defined “overweight” and “obesity” for both men and women.

**Figure 3.**
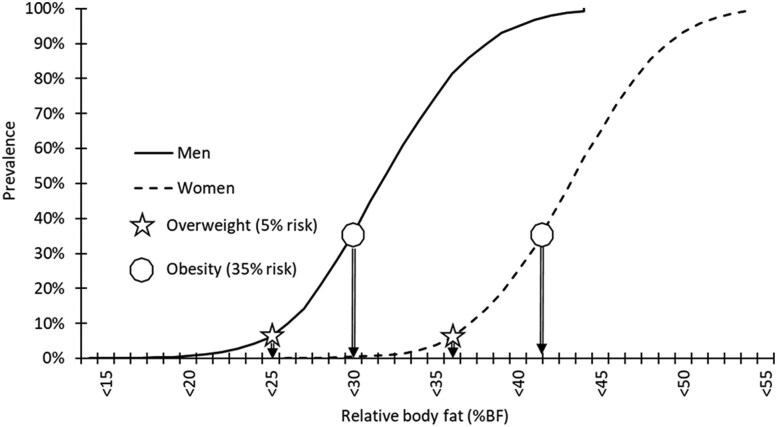
Prevalence of men and women in the NHANES data with ≥3 markers of metabolic syndrome by relative %BF.

The prevalence rates of MetSyn that corresponded to overweight and obese BMI thresholds (5% and 35%) were associated with 25% and 30%BF for men, and 36% and 42%BF for women, respectively (Fig. 3).

### Prevalence in Overweight and Obesity Based on Relative Body fat (%BF)

Based on conventional BMI thresholds, 29.5% of men and 33.1% of women were of healthy weight, 35.0% of men and 26.2% of women met the criteria for being overweight, and 35.5% of men and 40.7% of women for obesity. Using the %BF thresholds derived in this study, 27.7% of men and 27.2% of women had healthy weights, 34.0% of men and 33.5% of women were overweight, and 38.3% of men and 39.3% of women met the proposed %BF criteria for obesity.

### Relationship Between BMI and %BF

**Figure 4.**
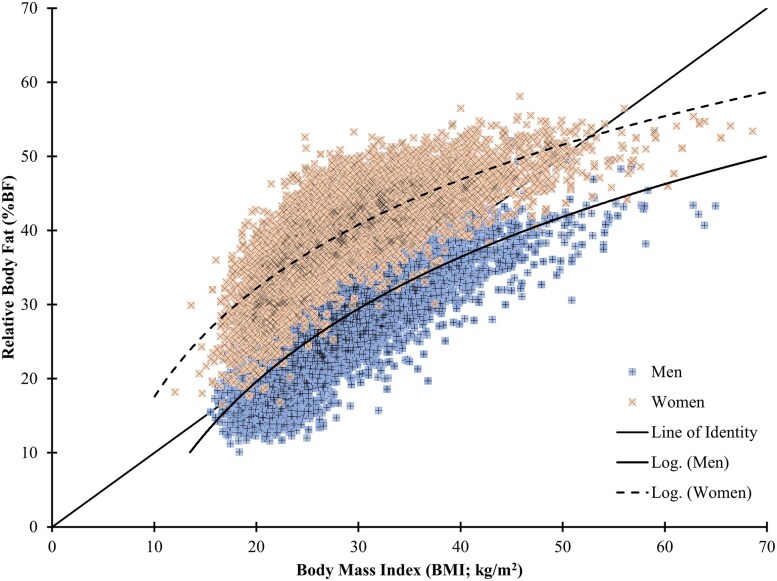
Comparison of BMI (kg/m^2^) to measured relative %BF for men and women in the NHANES data (1999-2018).

The BMI and %BF thresholds for MetSyn were independently established from the symptom-defined outcomes but similar agreement was observed for %BF predicted indirectly from BMI. Nevertheless, the poor predictive value of BMI for adiposity at the individual level was highlighted by the high scatter in a curvilinear relationship between BMI and %BF. The curvilinear relationship and the large scatter for individuals plotted by BMI vs %BF (Fig. 4) highlights one of the problems in using BMI to represent %BF. Although of BMI 25 kg/m^2^ and 30 kg/m^2^ correspond to mean %BF values very close to those associated with 5% and 35% prevalence of MetSyn in the current analysis, at an individual level, a different set of individuals will be identified at risk using the %BF metrics instead of BMI. Additionally, there is an obvious difference between how BMI to %BF tracks for men and women. This systemic difference in how BMI represents adiposity in women poses a number of potential problems when used to evaluate health outcomes.

## Discussion

Until recently, only anthropometric predictions of body composition were practical for population-wide obesity metrics. Direct assessments of %BF using emerging technologies such as multifrequency bioelectrical impedance (MF-BIA) are increasing in reliability and affordability, opening the door to more effective personalized obesity management. There is still a wide range in validity and precision of %BF-derived from bioelectrical impedance devices, methods, and predictive equations in comparison to criterion methods such as DXA (15-18). A large step in this direction is a policy-based adoption of these types of systems within the US Department of Defense for evaluation of their own body composition standards (15, 19, 20). This will surely also lead the way to wider use in clinical practices, with creation of epidemiological data with clinical outcomes to further refine %BF associations with disease. Receiver operating characteristic and area under the curve statistics were similar between BMI and %BF for both men (0.83 and 0.80) and women (0.75 and 0.71), highlighting sex differences in the general accuracy for both methods. In this study, the similarity of MetSyn receiver operating characteristic and area under the curve statistics for BMI and %BF indicate that there is more to be learned about the relationships between body composition and metabolic disease. Like BMI, the relative amount of stored fat may itself be only a proxy for more dynamic nutrition and exercise influences on metabolic disease as proposed by Blair et al (21, 22).

Obesity-related diseases may be more effectively managed by finally moving away from anthropometric estimations of adiposity to direct measurement of the fat component, generally expressed as relative fat (%BF) or fat mass index (fat mass/height^2^); we chose to use %BF in our analysis. This analysis suggests %BF thresholds for patient guidance; these metrics align with “overweight” (5% of MetSyn individuals) and “obesity” (35% of MetSyn individuals) in a large nationally representative sample. The %BF thresholds produced in this current analysis are in line with previously reported guideline estimates made from indirect analyses based on BMI relationship to %BF (2, 3). This is also relatively consistent with health-related %BF standards (26% for men and 36% for women) established by the US Army for the oldest category of soldiers (age 40+) on the basis of equivalence to BMI 25 kg/m^2^ for a large sample of soldiers assessed by underwater weighing in 1984 and 1985 (23).

Although BMI can be helpful as a first-level screening criteria, it is not an accurate method for determining body composition and, in fact, does not provide accurate information about fat and lean components. This point is well illustrated in Fig. 4, where the wide variation in the relationship between %BF and BMI is apparent for both men and women. This can be most problematic at both the lower and upper ranges of BMI because a person with an apparently healthy BMI (≤25 kg/m^2^) can have excess body fat (metabolically obese normal weight, “skinny fat”) (24, 25) and individuals with higher BMIs (>25 kg/m^2^) may carry high lean mass and not have excess relative fat (eg, athletes, military) (26-28). Further technological improvements in body composition assessments such as refinement of mathematical models for MF-BIA technologies to produce increasingly more accurate intraabdominal fat content may lead to the next generation of clinically relevant metrics of body health. Currently available methods such as DXA provide estimates of intraabdominal fat and other information about soft tissue lean mass and bone mineral content with emerging relationships to all-cause mortality (29), whereas MF-BIA systems also provide some of these other components of body composition with clinical utility and are continuing to evolve. Endorsement of body composition assessments by medical societies will help to encourage better insurance coverage to lower out-of-pocket expenses for patients and will facilitate greater use and application of these useful technologies.

## Data Availability

Data from this analysis is openly available to anyone under NHANES, found here: https://www.cdc.gov/nchs/nhanes/about_nhanes.htm.

## Abbreviations

%BF: percent body fat
BMI: body mass index
DXA: dual-energy x-ray absorptiometry
MetSyn: metabolic syndrome
MF-BIA: multifrequency bioelectrical impedance
NHANES: National Health and Nutrition Examination Survey
